# Modernizing Artisanal Brick Kilns: A Global Need

**DOI:** 10.1289/ehp.121-a242

**Published:** 2013-08-01

**Authors:** Charles W. Schmidt

**Affiliations:** **Charles W. Schmidt**, MS, an award-winning science writer from Portland, ME, has written for *Discover Magazine*, *Science*, and *Nature Medicine*.

The city of Cusco in southeastern Peru is a World Heritage Site and a tourist attraction for those drawn by the Inca ruins nearby. But anyone who lives in or visits Cusco has to contend with its heavily polluted air. Trucks and buses belch clouds of diesel exhaust, and on the outskirts of town hundreds of family-run artisanal brick kilns send plumes of oily black smoke into the sky. Brick makers near Cusco fire their traditional kilns mainly with coal, but they also have a long history of burning plastic trash, used motor oil, and discarded tires. “They produce huge air quality problems,” says Jon Bickel, a Swiss development expert based in Lima.

Most of the bricks that are made in Peru and other low-income countries are fired in artisanal kilns that pump greenhouse gases and other pollutants into the atmosphere, posing a health threat to brick workers, their surrounding communities, and even farther afield. And since brick production keeps pace with population growth, its environmental health impacts are likely growing throughout the developing world, says Jie Li, an energy and environmental specialist with the World Bank’s South Asia Region in Washington, DC.

**Figure:**
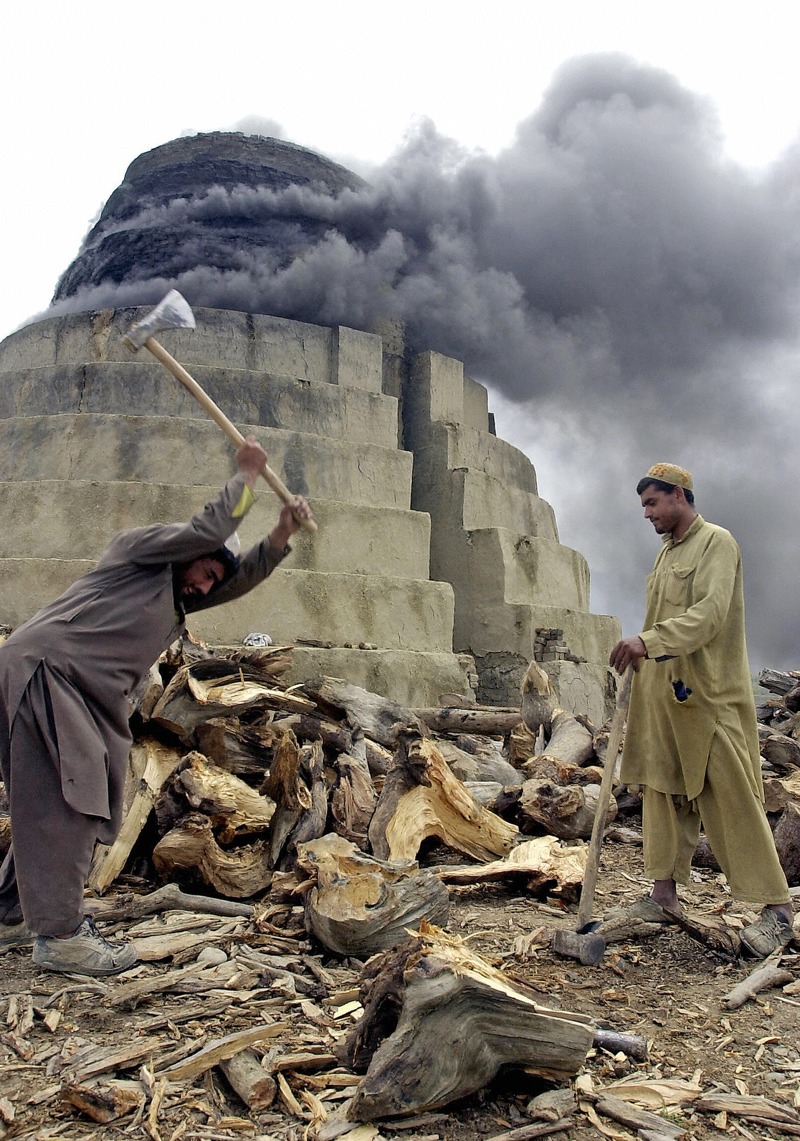
Workers cut firewood to light a brick kiln in Kabul, Afghanistan. The few health studies that have been conducted on kiln workers typically reveal musculoskeletal effects and health effects similar to those seen in other smoky, dusty occupations. © Shah Marai/AFP/Getty Images

A number of both small-scale and multinational development groups coordinated by the World Bank, the United Nations, and other organizations are now turning their attention to this important but poorly characterized industrial sector. Leading the charge in Latin America, Bickel directs a Swiss-funded group known by its Spanish acronym, EELA (for Eficiencia Energética en Ladrilleras Artesanales) that aims to modernize artisanal brick making in Peru, Argentina, Brazil, Bolivia, Ecuador, Colombia, Nicaragua, Honduras, and Mexico.

Bickel says EELA’s long-term goal is to reduce kiln emissions by 30–50%. But achieving that goal won’t be easy. Throughout Latin America and other countries in the developing world, artisanal kiln operators comprise an informal industry; they rarely pay taxes or obtain operating permits, and because of that, they often lack access to the financial credit and capital needed to purchase cleaner technology. Many also lack electricity (required for newer types of mechanized production) and access to roads and other infrastructure that could help in the modernization effort. Some are unwilling to change age-old manufacturing processes.

**Figure:**
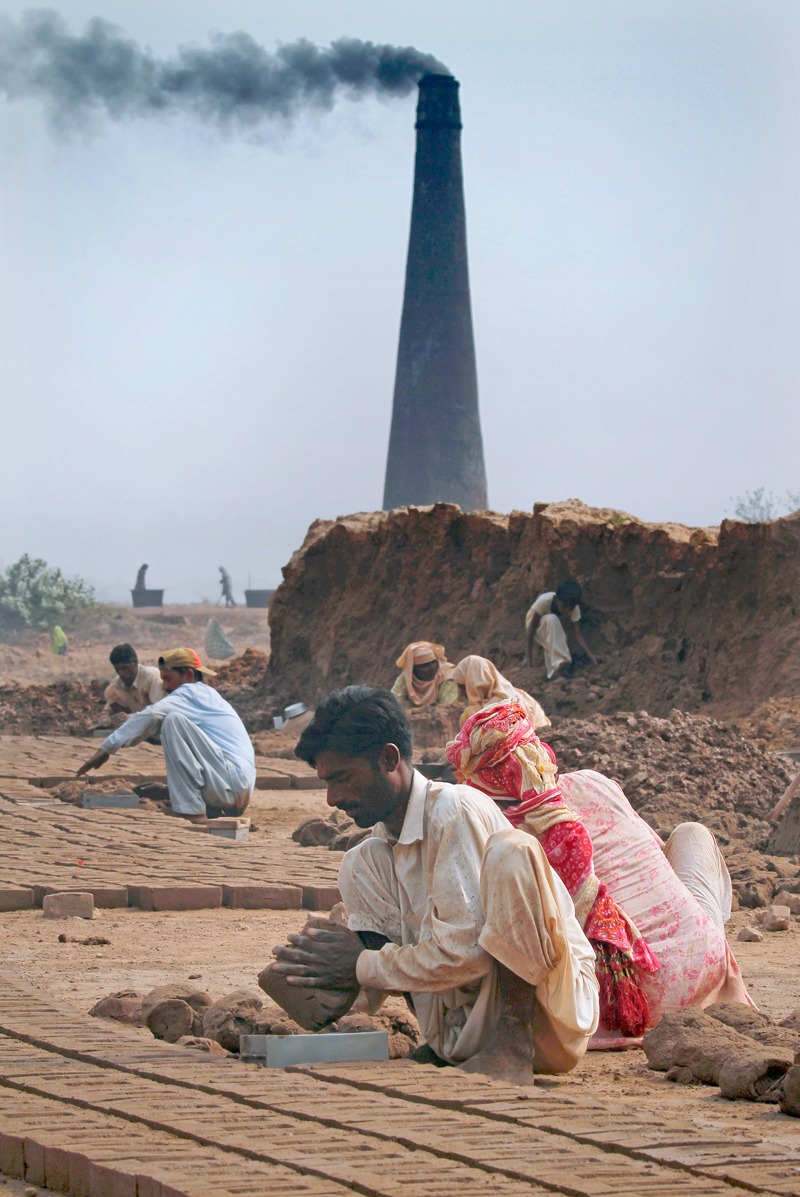
Pakistani families prepare bricks at a fixed-chimney Bull’s trench kiln on the outskirts of Islamabad. Under debt bondage, an entrenched custom in South Asia, families can work at kilns for years paying off loans from the owners. © AP Photo/Anjum Naveed

Still, the associated benefits of modernization justify the effort, Li says. In addition to the environmental health impacts associated with artisanal brick kilns, these operations employ “the poorest of the poor, including, in many instances, women and children,” Li says. “So by promoting more efficient kilns and manufacturing, we can also address related development problems, such as poverty, child labor, and women’s issues.”

## Modern versus Artisanal Brick Production

According to Ellen Baum, a senior scientist with the Boston-based nonprofit Clean Air Task Force, roughly 1.5 billion bricks are made worldwide every year. With an estimated annual output of 700–800 million bricks, China tops global production.^1^ But according to Li, Chinese manufacturing is increasingly dominated by modern technologies. These modern kilns produce lower emissions than those encountered in India, for instance, which is the world’s second largest-brick producer overall (and largest artisanal producer), or in Latin America, where producers tend to rely on the most primitive—and most polluting—types of kilns.

The more modern kilns used in China and developed countries rely on automated extruders, machines that churn out bricks like sausages on an assembly line. In contrast, artisanal bricks are shaped by hand—workers combine topsoil, manure, and other raw materials with water to make a thick slurry, which is then pressed into molds and dried in the sun. In the final steps, the sun-dried “green” bricks are fired in kilns and stacked on pallets for transport.

Greg Borchelt, president and chief executive officer of The Brick Industry Association in Reston, Virginia, says an automated factory equipped with pollution controls in the United States can make 30–100 million bricks a year with a workforce of 20–30 people. But a typical artisanal kiln in Bangladesh requires a workforce of 150 people to make 4 million bricks in a year.^2^ Latin American kilns tend to be much smaller than their Asian counterparts; Bickel says most of them are family run and produce bricks in batches as needed.

**Figure:**
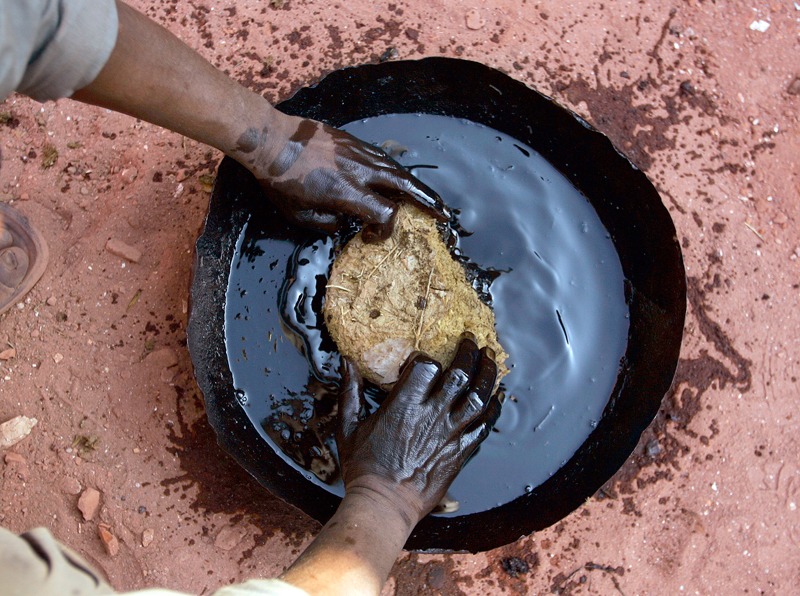
A worker soaks dry cow dung in a mixture of kerosene and gasoline at a kiln near Amritsar, India. The mixture will be used to ignite the kiln. © AP Photo/Altaf Qadri

**Figure:**
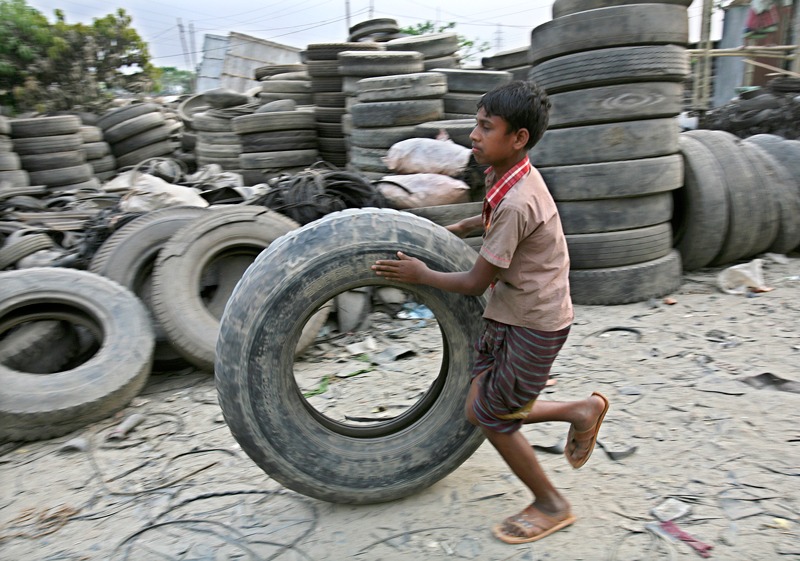
A boy transports a discarded tire in Dhaka, Bangladesh. Used tires and other trash have long been widely used as fuel for kilns, but some kiln owners are now using cleaner fuels. © Andrew Biraj/Reuters/Corbis

The few health studies of the sector that have been conducted link kiln work with respiratory disease and other problems such as musculoskeletal stress.^3^^,^^4^ One study, performed by investigators at Kathmandu Medical College, found that children living near brick kilns in Bhaktapur District, Nepal, were more likely to have upper respiratory infections, including tonsillitis and sore throat, than children living farther away from the kilns.^3^ Another study, by scientists at Aga Khan University in Karachi, Pakistan, reported high rates of chronic cough, phlegm, shortness of breath, wheezing, bronchitis, and asthma among both smoking and nonsmoking male brick workers.^4^ According to the authors, most of the findings were similar to health effects seen in workers in other smoky, dusty occupations.

Investigators with the International Justice Mission, a human rights organization in Washington, DC, have also found extensive evidence of “debt bondage” among artisanal brick workers in South Asia. This system involves workers and their families toiling for years in what can become a futile effort to pay back loans from kiln owners. Bickel says he hasn’t seen evidence of debt bondage in the Latin American brick sector. But given its family-run nature, child labor does occur in these countries, he says.

## Minimal Emissions Data

The sector’s air emissions are poorly characterized but may include sulfur oxides, nitrogen dioxide, carbon monoxide, carbon dioxide (CO_2_), forms of particulate matter (PM) including black carbon, and additional compounds released by the burning of coal and other fuels. Baum explains that emissions vary by kiln type and fuel burned, which makes it difficult to derive representative averages for the artisanal brick sector as a whole.

Investigators typically can’t rely on official data to estimate the number of kilns operating in specific areas; given their informal status, kiln operators don’t register with local municipalities. Bickel recalls that when EELA formed in 2010, neither governments nor local authorities could give accurate counts of the kilns in their jurisdictions. Of Cusco, he says, “They told us three to five kilns, but that was only the formal, registered enterprises. We had to go door to door to find out the true number, which was closer to three hundred.”

EELA has since calculated the emissions from Cusco’s kiln sector and found the amounts ranked relatively low, compared with other sources. But at a combined total of just over 1,000 metric tons, emissions of coarse particulate matter (PM_10_) from the Cusco kilns rank second to vehicular emissions, which—given that Peruvian transportation is dominated by diesel trucks and vehicles operating without pollution controls—is the country’s largest source of PM by far.^5^^,^^6^ Particulate pollution is a major health hazard that increases the risk of cardiovascular and lung disease.^7^

Elsewhere in the world, brick kilns are bigger sources of particulate pollution. Emissions of PM_10_ and PM_2.5_ (fine PM) from a cluster of roughly 530 kilns near the Bangladeshi capital of Dhaka are estimated to be responsible for 750 premature deaths from respiratory disease every year, or 20% of the total number of Dhaka residents who die annually as a result of poor air quality; in the dry season, when bricks are produced, kilns account for 38% of the PM_2.5_ in the city.^2^ “They become the number one source, greater even than the transportation sector,” says the World Bank’s Li.

**Figure:**
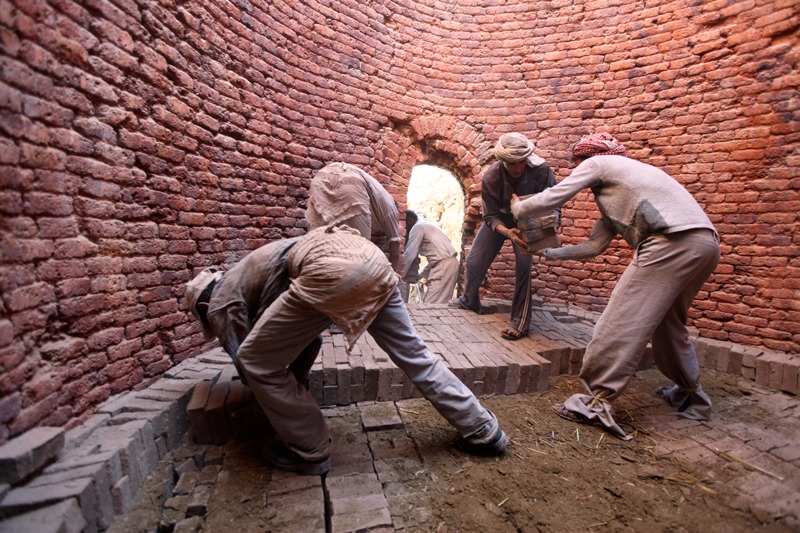
Workers stack green bricks inside an artisanal kiln in Sana’a, Yemen. Emissions vary by kiln type and fuel burned, which makes it difficult to derive representative averages for the artisanal brick sector as a whole. © Reuters/Mohamed al-Sayaghi

Modernization efforts aim to limit PM_10_ emissions to improve health, but they also seek to constrain CO_2_ and black carbon particles, which are important climate pollutants. Black carbon particles warm the atmosphere by absorbing and then radiating solar heat. In some instances they deposit on ice floes, darkening the ice and accelerating its melting. There is evidence that black carbon and other short-lived climate pollutants, such as methane and tropospheric ozone, are more potent than CO_2_ in terms of their influence on climate, but they also clear more rapidly from the atmosphere.^8^

Studies by the United Nations Development Programme and the World Bank suggest that premature death and diseases resulting from air pollution in Bangladesh could be cut in half by adopting cleaner kiln technologies.^2^^,^^9^ Similarly, it’s anticipated that new technologies could substantially reduce the sector’s climate impacts. EELA estimates that cleaner technology could reduce CO_2_ emissions from Latin American kilns by at least 30–45%.^10^

Artisanal kilns have caught the attention of projects aimed at climate change mitigation. A new report published by the International Global Atmospheric Chemistry Project, based at the University of Colorado Boulder, puts artisanal brick production on a list of priority targets for black carbon reduction.^10^ And the Climate and Clean Air Coalition to Reduce Short-Lived Climate Pollutants, which was launched in 2012 and now has backing from 31 international governments, is focusing on brick production as a key area for making substantial reductions in emissions of short-lived climate pollutants.^12^

## Efficiency Improvements

Just as the scale of artisanal brick making varies in different countries, so does the task of modernization. Latin American producers using the most primitive kilns are in essence starting from scratch, whereas for Asian producers the goal is to boost the efficiency of kilns that already use more sophisticated designs.

An early design improvement came during the mid-1800s, when the British engineer William Bull invented the Bull’s trench kiln (BTK) that bears his name. Bull, noticing that kilns’ appetite for fuel was denuding the Indian landscape of trees, designed the BTK to take advantage of waste heat from newly fired bricks and fuel combustion.^13^ The kiln consists of a circular or oval trench in which green bricks are stacked in a lattice pattern. Workers create a continuous firing zone—they shift the fires and moveable metal chimneys from one section of the trench to the next, meanwhile steadily replenishing the supply of green bricks and removing finished ones.

Measuring roughly 30–50 feet high, these chimneys do not produce sufficient draft to ensure the complete combustion of fuel, and they release emissions near the ground. So in 1996 the Indian government banned BTKs in favor of a newer design outfitted with a single chimney roughly 120 feet high. By increasing the draft and emitting pollutants higher in the sky, the so-called fixed-chimney Bull’s trench kiln (FCBTK) helps reduce worker exposures. It is now used by 40% of India’s brick makers, accounting for 70% of the country’s total brick production.^14^ However, like the original BTK, the FCBTK exposes workers to thick, dusty ash when they unload bricks from the kiln, according to Sameer Maithel, founder and director of the New Delhi–based consulting firm Greentech Solutions.

**Figure:**
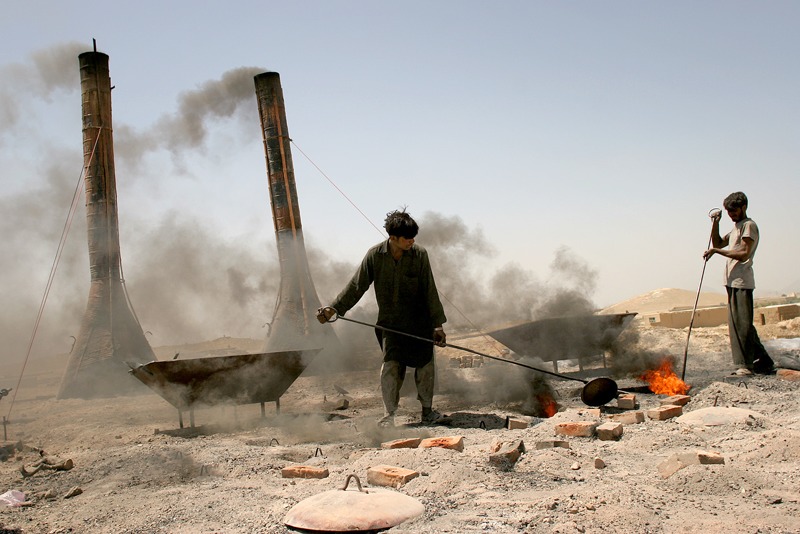
Workers pour coal into the fire of a Bull’s trench kiln outside Kabul. Continual kilns like this can operate without stopping for months and even years as workers steadily fire fresh batches of bricks. © AP Photo/Tomas Munita

China, meanwhile, relies on two even more efficient kiln designs. The Hoffman kiln is a house-like structure consisting of a main firing passage in which the fire travels through chambers full of green bricks—it is essentially a BTK with a permanent roof. In the other design, known as a tunnel kiln, conveyors loaded with bricks travel through a long, straight passage. The bricks are gradually heated to firing temperature, then cool down as they exit. With significantly reduced exposure to dust and heat, Maithel says the tunnel kiln offers the best working conditions for workers.

Unlike these more permanent kilns, which can run continuously for months or even years at a time, the traditional kilns that dominate Latin American production are used to fire bricks in batches. The kilns come in different varieties, but in general they amount to little more than piles of bricks in different configurations, stacked to maximize internal airflow between them. The kilns generally cost no more than US$1,000 for a simple enclosure. But because they’re inefficient, they use a lot of fuel. And from EELA’s perspective, high fuel costs offer a market opportunity: Artisanal producers will pay to upgrade their manufacturing processes and improve the quality of their products, Bickel says, if doing so saves them money over time.

## Exploring Modernization Options

The main barriers to adopting new technologies, Li says, are cost and lack of knowledge. Upgrades can run into the tens of thousands of dollars, if not much more, while cleaner, industrial-scale Hoffman or tunnel kilns can cost millions. Most kiln operators don’t have the capacity or incentive to make these changes, Li says: “At the end of the day, producers need access to credit, but since they aren’t recognized as formal entities, banks see them as risky clients.”

According to Bickel, EELA’s development efforts rely heavily on market forces. “A government or group that pays for new kilns might purchase only forty or fifty,” he says. “But in Latin America, we have close to forty thousand kilns. So we’re trying to boost incentives and offer financial opportunities that allow producers to upgrade on their own. Otherwise, we have no chance of improving the sector.”

EELA validated a number of options for improving kiln efficiency. It ultimately settled on a multipronged modernization strategy that starts with the simplest of measures: a fan that blows air at smoldering kiln fires to boost their combustion. According to Jorge Luis Delgado, EELA’s financial specialist, the US$1,200 fan reduces fuel needs by 30%. The fuel savings, he adds, leads to an equivalent 30% reduction in CO_2_ emissions while brick production rates stay constant. “When we started the project, there was only one vendor selling the fans in Cusco,” Bickel says. “Now we have five vendors, and nearly all the brick producers are using them.”

In its second phase, EELA aims to promote the fan in combination with a more efficient “downdraft” kiln that edges CO_2_ reduction and fuel savings closer to 50%. Smaller than an industrial-sized Hoffman or FCBTK kiln, the downdraft model also recycles heat to warm prefired bricks. The cost of the kiln, which can be built using local materials, ranges up to US$10,000. That investment, Delgado says, can be partially offset by savings from using a fan with a traditional kiln. Bickel says the fans typically pay for themselves in about 6 months, whereas the kilns take about 2–3 years.

**Figure:**
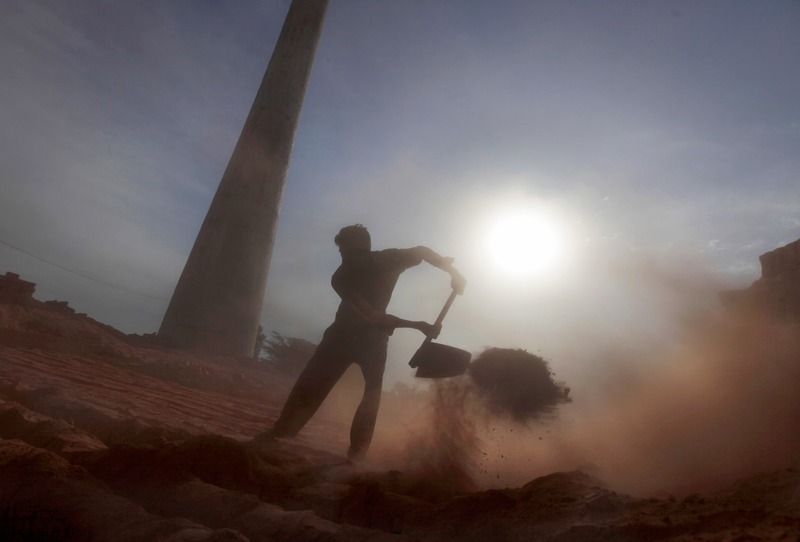
A worker in Amritsar removes sand from a batch of fired bricks. Although fixed-chimney kilns like this are somewhat cleaner than Bull’s trench kilns, the work is still plenty dirty. © AP Photo/Altaf Qadri

Despite the savings, the use of a fan can’t cover the cost of a downdraft kiln, so EELA works with financial entities that wouldn’t ordinarily loan to informal brick makers, such as local microfinancial institutions. Such institutions will supply microloans at lowered interest rates, Delgado says, but only if they’re convinced that more efficient kilns will boost producer incomes and lessen their own financial risk. EELA aims to supply evidence to convince them of that. The program is also coordinating with representatives from a new program known as EcoMicro, which was established in 2012 by the Multilateral Investment Fund in cooperation with the Nordic Development Fund, to create microfinancing options for borrowers in Latin American and Caribbean countries.

The World Bank, meanwhile, supports other strategies for brick modernization in South Asia, particularly in Bangladesh and India. These include mixing the clay used to make bricks with coal to enhance firing, piloting cleaner kiln technologies, demonstrating alternative building materials (e.g., hollow and perforated bricks require less raw material and energy to fire without sacrificing strength), and automating the laborious mixing and extrusion processes used in brick production.^2^

The Bank is trying to ease investment barriers in two ways. First, it acts as a trustee for carbon credits sold by brick producers in South Asia through a Clean Development Mechanism sponsored by the United Nations Framework Convention on Climate Change. Second, it finances the Bangladeshi government’s efforts to provide technical support to polluters who want to adopt cleaner methods. This latter effort occurs through the Bank’s Clean Air Sustainable Environment Project.

**Figure:**
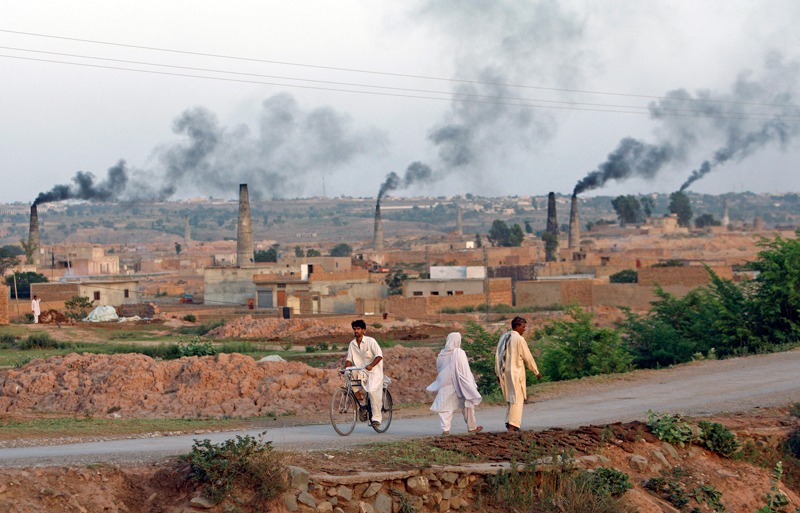
Brick kilns dot the landscape in Rawalpindi, Pakistan. A study published in 2012 showed that hydrogen fluoride emissions from kilns in nearby Peshawar severely damaged certain economically important crops.^18^ © AP Photo/Dita Alangkara

In Bangladesh, newer kiln models promoted through that project reduce particulate emissions by up to 80% and cut fuel needs—and commensurately, CO_2_ releases—by half.^2^ And in India, manufacturers can earn carbon credits for producing bricks that are compressed instead of fired, so no coal is used.^15^ Moreover, in 2012 the Asian Development Bank approved a $50-million credit line to support investments in cleaner and energy-efficient brick production in Bangladesh.^16^

## The Role of Governments

Maithel says national governments play an important role in modernizing brick production. Indeed, the Chinese and Vietnamese governments, both deeply involved in their respective building sectors, strongly promoted the use of tunnel kilns for brick firing. More recently, China banned solid bricks in urban areas in favor of equally strong hollow bricks. In China’s case, this was driven by food concerns as well as worries about air quality; the topsoil used as a raw material for bricks subtracts from the soil that’s available for planting.

Maithel suggests that by tightening its national FCBTK particulate emissions standard, the Indian government could promote what he says is a win–win proposition for the artisanal brick sector: retrofitting FCBTKs with “zigzag” modifications that alter the path of flue gases in the kiln (so they gain velocity, improve combustion, and transfer more heat to bricks as they fire), which could cut fuel needs and CO_2_ emissions by 20%.^17^ Baum says new, unpublished measurements confirm these modified kilns also emit less black carbon. Costing up to US$45,000, zigzag modifications have a payback time of 1–3 years, and offer an alternative to the much more expensive Hoffman and tunnel kilns.

**Figure:**
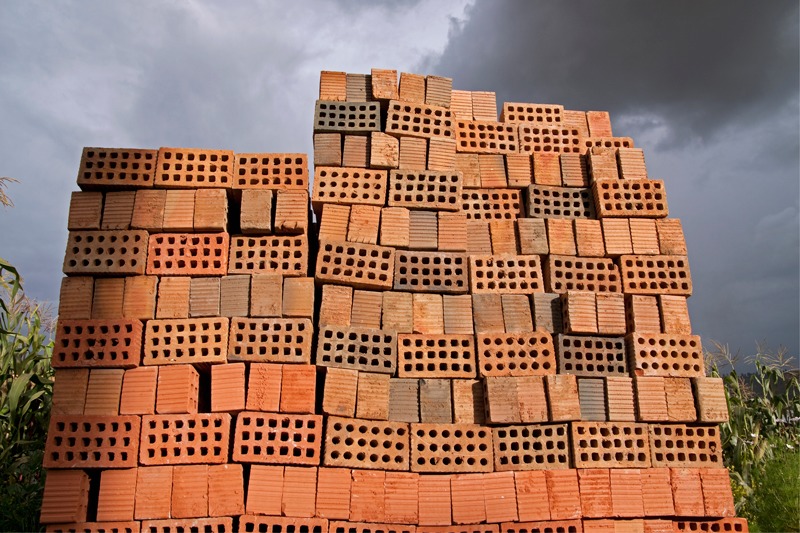
These bricks were made in a more environmentally friendly oven in Cusco, Peru. EELA, a Swiss-funded development group working in Cusco and other Latin American locales, hopes to reduce the region’s kiln emissions by 30–50% over the long term. © Michael Langford/Getty Images

The Indian government could also adopt policy measures such as prohibiting construction of new FCBTKs, Maithel suggests. But apart from its move to ban the moveable BTK, he says the Indian government has been slow to take interest. “My sense is that the government has many environmental priorities and may feel it is very difficult to regulate such small enterprises that are scattered all over the place,” he explains. “But the government also doesn’t recognize artisanal brick making as an important employment-generating sector, which provides employment to more than ten million workers. Working conditions are tough, but it provides a basic building material and employment in a poor country.”

Bickel agrees that, by formalizing the sector, governments could go a long way toward reducing its environmental impacts. In the meantime, the nascent effort to modernize traditional brick making—promoted by a small, but dedicated group of individuals—is gathering momentum. “We’re seen a lot of rapid progress,” Bickel says. “When brick producers see how quickly they can get a return on investment, almost all of them move to adopt new kiln technology.”
